# Immune Development and Intestinal Microbiota in Celiac Disease

**DOI:** 10.1155/2012/654143

**Published:** 2012-09-11

**Authors:** Tamara Pozo-Rubio, Marta Olivares, Esther Nova, Giada De Palma, Jorge R. Mujico, Maria Desamparados Ferrer, Ascensión Marcos, Yolanda Sanz

**Affiliations:** ^1^Immunonutrition Research Group, Department of Metabolism and Nutrition, Institute of Food Science, Technology and Nutrition, Spanish National Research Council (ICTAN-CSIC), Calle José Antonio Novais, 10, 28040 Madrid, Spain; ^2^Microbial Ecology and Nutrition Research Group, Institute of Agrochemistry and Food Technology, Spanish National Research Council (IATA-CSIC), Avenida Agustín Escardino, 7. Paterna, 46980 Valencia, Spain

## Abstract

Celiac disease (CD) is an immune-mediated enteropathy, triggered by dietary wheat gluten and similar proteins of barley and rye in genetically susceptible individuals. The etiology of this disorder is complex, involving both environmental and genetic factors. The major genetic risk factor for CD is represented by HLA-DQ genes, which account for approximately 40% of the genetic risk; however, only a small percentage of carriers develop the disease. Gluten is the main environmental factor responsible for the signs and symptoms of the disease, but exposure to gluten does not fully explain the manifestation of CD. Epidemiological and clinical data suggest that environmental factors other than gluten might play a role in disease development, including early feeding practices (e.g., breast milk *versus* formula and duration of breastfeeding), infections, and alterations in the intestinal microbiota composition. Herein, we review what is known about the influence of dietary factors, exposure to infectious agents, and intestinal microbiota composition, particularly in early life, on the risk of developing CD, as well as the possible dietary strategies to induce or increase gluten tolerance.

## 1. Introduction

CD is an immune-based enteropathy triggered by dietary wheat gluten and similar proteins in barley and rye in genetically susceptible individuals. The histological features of CD are villous atrophy, crypt cell hyperplasia, and increased number of intraepithelial cells. It is generally accepted that CD is a T-cell mediated disease, in which gliadin-derived peptides activate lamina propria, infiltrating T lymphocytes. This leads to the release of proinflammatory cytokines, such as IFN-*γ* and IL-15, which are responsible for the activation of the cytotoxicity of intraepithelial lymphocytes that leads to a profound tissue remodeling [1, 2].

This is a complex disorder, with environmental and genetic factors contributing to its etiology. The main genetic influence on CD is the HLA locus [3], specifically MHC class II genes that encode HLA-DQ2 (HLA-DQ2.5 and HLA-DQ2.2) and HLA-DQ8 heterodimers. The strongest association is with HLA-DQ2.5 heterodimer. The risk heterodimer HLA-DQ2.5 can be encoded in *cis*, when both DQA1*0501 and DQB1*0201 alleles are located on the same haplotype, or in *trans*, when these two molecules are located on different haplotypes [4]. HLA-DQ genes account for approximately 40% of the genetic risk. Around 90–95% of CD patients express HLA-DQ2 heterodimer while the remaining 5–10% patients express the HLA-DQ8 heterodimer [2]. However, it is still unknown why only a subset of individuals expressing HLA-DQ2 and HLA-DQ8 heterodimers develop CD, and why some do so very early in infancy after their first exposure to gluten, and others in adulthood [2, 5]. Thus, HLA-DQ2 and HLA-DQ8 heterodimers underlie the disorder but are not sufficient alone, thus other genetic and environmental factors must be involved in CD onset [5].

To date, gluten is the only known environmental factor to play a direct causal role in CD, and the only treatment for CD is a gluten-free diet (GFD). Wheat-gluten proteins include gliadins and glutenins. The closely related proteins in barley and rye that activate CD are hordeins and secalins, respectively. The gliadins are subdivided into *α*/*β*, *γ*, and *ω*-gliadins, while the glutenins consist of low molecular weight (LMW) and high molecular weight (HMW) glutenins. Gluten has high concentrations of glutamine and proline residues (35 and 15% of the total amino acid content) [6]. High proline content renders these proteins resistant to complete proteolytic digestion by gastric, pancreatic, and brush border enzymes in the human intestine, since these enzymes are deficient in prolylendopeptidase activity [7, 8], making it possible for large immunogenic gluten peptides to accumulate and reach the mucosal surface [8, 9]. While gluten is the main environmental factor responsible for the signs and symptoms of CD, other environmental elements might play a role in disease onset, either on their own or by interaction with gluten exposure.

### 1.1. Prevalence and Epidemiology

The first publication to demonstrate the true magnitude of CD was published by Catassi et al. in 1996, reporting a CD prevalence of 1 : 184 in an Italian scholar population and a ratio of known to undiagnosed CD cases of 1 in 7 [10]. Although screening studies predict CD to affect approximately 1% of the US population, including both children and adults, only 10–15% of these individuals have been diagnosed and treated [11]. An international serological screening study revealed differences in populations across Europe with high values in Finland (2.4%) and low values in Germany (0.3%), while prevalence recorded in Italy was 0.7% [12]. An analysis by Abadie et al. [4] showed there are no significant correlations between CD prevalence worldwide and wheat consumption, or between the sum of DR3-DQ2 and DR4-DQ8 frequencies, or the product of both factors. This would suggest that other environmental and genetic factors must contribute to the development or pathogenesis of CD. Due to the heterogeneity of CD manifestations, epidemiologists refer to the clinical and pathological spectrum of the disease as an iceberg, including active, silent, latent, and potential CD [4, 10, 13].

### 1.2. Immunopathology of Celiac Disease

#### 1.2.1. Adaptive Immune Response: Gluten and HLA-DQ Molecules

It is well known that gluten specific CD4+ T cells can be isolated from CD patients but not from healthy individuals [14]. In CD patients, gluten-derived peptides are recognized by HLA-DQ2 or HLA-DQ8 heterodimers of antigen-presenting cells, which trigger a CD4+ T cell response. These two HLA-DQ molecules have preference for negatively charged peptides. Although native gluten peptides lack negative residues, the tissue transglutaminase 2 (TG2) can convert noncharged glutamine into negatively charged glutamic acid, a process called deamidation. Deamidated gluten peptides are presented to CD4+ T cells with subsequent release of proinflammatory cytokines, such as IFN-*γ* that enhances activation of cytotoxic CD8 intraepithelial lymphocytes, contributing to a profound tissue remodeling.

TG2 is mostly retained intracellularly in an inactive form and is activated upon its release during tissue damage; however, the question of how TG2 is converted to its active form is still unclear. Tjon et al. [15] suggest that CD4+ T cells could respond against native gluten peptides representing the first breach in oral tolerance to gluten. Activated gluten-specific CD4+ T cells can also stimulate B-cell production of antigluten, as well as anti-TG2 antibodies [16]. In 1970, Shiner and Ballard [17] were the first to report IgA deposit in the basement membrane of surface epithelial cells, in crypt epithelium, around the subepithelial fibroblast and in the walls of blood vessels in the intestinal mucosa of celiac patients, later corroborated by other studies [18, 19]. IgA deposits have also been found in skin and brain promoting dermatitis herpetiformis [20] and gluten ataxia, respectively [21]. However, whether IgA antibodies against either gluten or the autoantigen TG2 are byproducts of the intestinal adaptive immune response or whether they play a direct role in CD pathogenesis remains unclear [2].

Recently, Matysiak-Budnik et al. [22] hypothesized a transport function for antigliadin IgA antibodies. They proposed that gluten peptides may be complexed to intraluminal secretory IgA, bound to an IgA receptor and transported, protected from lysosomal degradation by a specific transcytosis pathway. The transcytosis of IgA in CD seems to involve the transferrin receptor CD71, since in active CD, CD71 expression is increased and CD71 is found at the apical enterocyte membrane where it colocalizes with IgA. By contrast, in the normal intestine and in patients on a gluten-free diet, CD71 is only expressed on the basolateral enterocyte membrane.

#### 1.2.2. Intraepithelial Lymphocytes: Between Adaptive and Innate Responses

Most IELs are CD8+ TCR*αβ*+ T cells (75% CD8+ TCR*αβ* and 15% TCR*γδ*+ T cells) [2]. In active CD the number of IELs (CD8+ TCR*αβ*+ and TCR*γδ*+ T cells) is increased. In active CD, IELs express high levels of activating receptors like CD94/NKG2C and NKG2D [23] and intestinal epithelial cells have increased expression of CD94/NKG2C and NKG2D ligands (MICA and HLA-E) [24]. The interaction of NK receptors with their ligands leads to the death of intestinal epithelial cells and releases IFN-*γ* and cytolytic proteins (perforin, granzymes, etc.), resulting in observable tissue damage. IL-15 has been shown to upregulate both CD94/NKG2C and NKG2D NK receptors in IELs of active patients, boosting their ability to lyse enterocytes [15, 24].

#### 1.2.3. Innate Immune Response

Some gluten peptides can induce tissue damage by directly activating components of innate immunity [25]. The peptide p31-43/49 has been shown to activate the production of IL-15 and the NK-receptor-mediated cytotoxicity by IELs, independent of TCR specificity [26]. The presence of a receptor for p31-43/49 in intestinal epithelial cells has not been found yet and, thus, the molecular mechanism underlying the biological effects observed for this peptide remains unclear [15].

## 2. Influence of Dietary Factors on Immune Development in a CD Context

Dietary factors affecting disease risk in later life seem particularly relevant at early stages when the immature neonate's gut is acquiring and shaping its own microbiota and undergoing major physiological and immunological developments up to the point when the immune system acquires full competence and tolerance to nonharmful antigens [27].

Infants' first exposure to dietary gluten seems particularly important in defining the risk of developing CD in predisposed subjects. The effect of timing of gluten introduction on CD was first reported at the end of the 1970s by pediatricians in West Somerset, UK [28]. The authors noticed that the incidence of CD declined from 1 : 1228 to 1 : 4168 following the recommendations to avoid both the addition of cereals to bottle feeds and the introduction of gluten before 4 months of age. The role of age at the first gluten exposure in CD onset was later studied by Norris et al. [29]. They carried out a prospective observational study from 1994 to 2004 in 1560 children with an increased risk of CD or type I diabetes, as carriers of either HLA-DR3 or DR4 alleles or as having a first-degree relative with type I diabetes. In these children they assessed the risk of CD autoimmunity (CDA) defined as being positive for tissue transglutaminase (tTG) autoantibody in two or more consecutive visits. The mean followup was 4.8 years. Infants exposed to gluten in the first three months of life have a 5-fold increased risk of autoimmunity compared with infants first exposed at 4–6 months (hazard ratio (HR) 5.17, 95% CI 1.44–18.57). Infants introduced to gluten at 7 months or later also had an increased risk of CDA compared with those exposed between 4 and 6 months (HR 1.87, 95% CI 0.97–3.00). This study did not find any evidence for a protective effect of prolonged breastfeeding. The median duration of breastfeeding was 5 months both in CDA positive and in CDA negative children. This analysis, however, was not restricted to HLA-DR3 children and possibly the protective effect of breastfeeding was evident only when children with genetic risk were considered. The discrepancy between the results of this study and others reporting the protective role of breastfeeding might be explained by the different methodologies between retrospective and prospective studies. Another factor to consider is the different dietary practices in Europe and the United States [29], since in Europe gluten tends to be introduced as a replacement of breast milk at weaning (e.g., the flour-based follow-up infant formula once used in Sweden) whereas in the United States this does not seem to be the case. Some explanations have been reported by Norris et al. [29] for the increased risk of CDA when first gluten exposure occurs in younger and older children instead of at the age of 4–6 months. On the one hand, in younger children, early introduction of solid foods (i.e., before the intestinal immune system reaches a certain level of maturation) may lead to intolerance [30]. On the other hand, in children aged 7 months or older, the factor leading to the increased risk of CDA might be the large amounts of gluten intake at the first exposure [29].

A position paper by the ESPGHAN Committee on Nutrition has outlined possible practical suggestions on the introduction of complementary feeding to avoid both early (<4 months) and late (≥7 months) introduction of gluten, and to gradually introduce small amounts of gluten whilst the infant is still breast-fed [31]. This change in the policy of complementary feeding is aimed at modulating the predisposition of chronic disorders later in life (particularly of CD) and has already been adopted by countries like Australia [32]. This is, however, still a matter of debate since exclusive breastfeeding for around six months is considered a desirable goal both by ESPGHAN [33] and the WHO, in order to support healthy growth and development and reduce the risk of infections. Furthermore, the evidence for beneficial effects of delaying the introduction of complementary foods until 6 months is very limited, and difficult to support in the face of emerging evidence that it may be detrimental [34]. As suggested by Agostoni and Shamir [31] perhaps the 6-month theorem should be partly revised and small amounts of solids, including gluten, be allowed in the 4 to 7-month temporary window to modulate the genetic predisposition towards an autoimmune response, especially in developed countries were the exposure to infectious agents differs from that in the developing countries and, therefore, the risk of infections. However, the time of first exposure to potentially allergenic foods in infants differs significantly between countries, and they are introduced much earlier than recommended in some countries [35]. Moreover, formula-fed infants receive potentially allergenic foods earlier than breast-fed infants [35].

The protective role of breastfeeding against CD onset has been reviewed and analyzed in a meta-analysis of observational retrospective studies published in May 2004, which concluded that increased duration of breastfeeding is associated with a reduced risk of CD [36]. Five of the six case-control studies satisfying the inclusion criteria of methodological quality, found that children with CD had been breastfed for a significantly shorter period compared to controls. Age at the time of risk evaluation was variable among participants and among studies, ranging from less than 2 years to a median of 7.9 years. Also, the meta-analysis of four of these studies led to the conclusion that the risk of developing CD was significantly reduced in children who were breastfed at the time of gluten introduction (OR 0.48, 95% CI 0.40 to 0.59). However, the reviewed studies do not clarify whether breastfeeding only delays the onset of symptoms or provides permanent protection against the disease. Furthermore, the results of the meta-analysis are subject to limitations such as “recall bias” whereby the duration of breastfeeding and the age of first gluten exposure may be mistaken, or another bias might be derived from suboptimal adjustment for potential confounding factors across children who were breastfed and those who were not. For instance, only one of the studies controlled the HLA genotype and this was the only study that did not find a relationship between breastfeeding and protection against CD; however, since this was a very small study registering only eight cases of children with CD, a type II error was likely to have occurred.

The study by Ivarsson et al. [37], included in the meta-analysis, is a population-based incident case-referent study of 627 cases with confirmed CD (reported to a CD register between November 1992 and April 1995) and 1254 referents assessing patterns of complementary food introduction to infants. The study revealed that the risk of CD was reduced in Swedish children aged <2 years if they were still being breast-fed when dietary gluten was introduced (adjusted OR: 0.59; 95% CI: 0.42–0.83) and the risk increased when the gluten was introduced in the diet in large amounts (OR: 1.5; 95% CI: 1.1–2.1). In this study the effect of age at the time of gluten introduction was not conclusive. It is biologically likely that the presence of breast milk at the time gluten is introduced increases the chance of developing oral tolerance for the antigens of importance. It is unclear whether this possibility is only effective during a certain period in infancy. In the aforementioned study, the exposure risk factors explored were of no or only minor importance in children older than 2 years. Therefore, it is also important to pursue further whether favorable infant dietary patterns postpone CD onset or in fact reduce the overall lifetime risk of the disease [37].

Previous to this analysis of risk factors, the authors had documented an epidemic of symptomatic CD between 1984 and 1996 in Swedish children under 2 years of age, partially explained by changes in infant feeding [38]. The increased incidence was preceded by an increase in the amount of gluten consumed and by delayed exposure to dietary gluten, which might have resulted in a higher proportion of children being introduced to large amounts of gluten after breastfeeding was withheld. Recently, a Swedish study of the prevalence in children born in 1993, during the epidemic, and under these unfavorable dietary practices, revealed that the prevalence of CD at 12 years of age was as high as 3%, 3-fold higher than the commonly reported prevalence of 1% [39].

Other recent studies have pointed to the role of breastfeeding in delaying CD in infancy. D'Amico et al. [40] showed that children with CD who had been exclusively breastfed had a delayed onset and less severe disease symptoms than those who had not been exclusively breastfed [40]. Medical records of 89 infants (7–14 months of age) diagnosed with classic CD at the University Children's Hospital in Belgrade, from 2000 to 2008, were retrospectively analyzed to determine the duration of breastfeeding and timing of gluten introduction [41]. Breastfeeding, although generally short, significantly reduced the risk of CD onset in the first year of life (OR 0.655; 95% CI 0.48 to 0.89); moreover, the duration of breastfeeding was the most significant predictor of developing CD (*B* = 0.49; 95% CI 0.13 to 0.77). One-fourth of the infants were being breastfed at the time of gluten introduction, and they developed the disease later than the infants who were not being breastfed at the time of gluten introduction. This finding supports the protective effect of consuming human milk while introducing other food antigens [41]. However, this study also poses the question of whether the action of breastfeeding might not indirectly delay the onset, the symptoms, and the diagnosis due to a delay in gluten exposure in breast-fed infants.

In spite of all the evidence reported, it remains unclear whether those children breast-fed during the introduction of gluten are more likely to develop an extraintestinal (“atypical”) CD [42]. A series of 162 celiac children registered by the University of Chicago, revealed that children breast-fed at the time of gluten introduction were just as likely to develop intestinal as extraintestinal symptoms, whereas children who were not breastfed when weaned with gluten had a much higher chance of developing intestinal symptoms [43].

Given the gaps in knowledge and the limitations of the studies described above, it seems clear that long-term prospective cohort studies are required to investigate further the relationship between breastfeeding and CD.

The following hypotheses have been formulated to understand the protective role breastfeeding plays in CD onset.

- Breastfeeding might affect tolerance induction in infants because of both the possible transfer of small amounts of gluten and gluten-specific IgA antibodies through breast milk and the presence of factors in breast milk that affect immune system maturation and responses [44]. Human milk provides many bioactive factors, including antimicrobial and anti-inflammatory agents, enzymes, hormones, and growth factors, many of which are involved in gut maturation and development of the infant's innate and acquired immunity [45, 46]. A particular feature of the neonatal immune system is the presence of a wide pool of naïve T cells waiting to participate in primary immune responses. Intestinal antigen exposure during neonatal life influences the T cell receptor (TcR) repertoire of these developing immature T cells [47]. In our recent PROFICEL study the combined effect of HLA-DQ genotype and milk-feeding practices at 4 months of age on peripheral lymphocyte subsets was studied in a group of infants with at least one first-degree relative suffering from CD. Our analysis revealed an independent effect of milk-feeding practices on specific T cell subsets. The results showing a higher percentage of T CD4+CD25+ cells and lower T CD4+CD38+ counts in breast-fed than in formula-fed infants, suggest that breast-fed infants could have a more mature immune system than formula-fed infants [48]. Further research is now underway to ascertain whether the lymphocyte subset profile of breast-fed infants is associated with a better response to gliadin after gluten introduction.
- Children that are breastfed when gluten is introduced during weaning, receive lower amounts of gluten, even on a protein/kg body weight basis [42].
- As suggested from studies showing a reduced risk of childhood type I diabetes with breastfeeding and late introduction of cow's milk, it seems plausible that in addition to the immunologically active components in breast milk, avoidance of the early introduction of cow's milk protein contributes to the protective effect of breastfeeding [31]. This might also be the case for the role of breastfeeding in CD prevention.
- Other reasons that could explain a protective effect of breastfeeding against CD development could be related to the role human milk plays in defining microbiota composition [49] and in the incidence of infections, which is explained in the next section.

## 3. Intestinal Microbiota and CD

The composition of the intestinal microbiota has also been related to CD recently. The first associations were established in CD patients, untreated and treated with a gluten-free diet [50–52]. The intestinal dysbiosis of CD patients was characterized by increases in numbers or proportions of *Bacteroides* spp. and reductions in those of *Bifidobacterium* spp. and *B. longum*, which were not completely normalized after patient adherence to a GFD [50–52]. *E. coli *and* Staphylococcus* numbers were also higher in feces and biopsies of untreated CD patients than in those of controls, but the differences were normalized after gluten withdrawal [51]. The analyses of the prevalence of bacterial species associated with duodenal biopsies also revealed a reduction in *Bacteroides* species diversity in patients, untreated and treated with the GFD, in comparison with controls [53]. In addition, *E. coli* clones isolated from untreated and treated CD patients carried a higher number of virulence genes than those from healthy children. These findings indicated that intestinal dysbiosis is associated with CD and that some of the alterations are not only secondary to the inflammatory milieu of the active phase of this disorder, but might play a primary role together with genetics and gluten intake in this disorder.

Further studies are ongoing to establish the possible effects of milk-feeding patterns and HLA-DQ genotype on the microbiota in early life, and the possible influence on later development of CD [54, 55]. In this context, a prospective study including 164 healthy newborns, with at least one first-degree relative with CD, indicated that both the milk-feeding type and the HLA-DQ genotype play a role in establishing infants' gut microbiota [55]. Reduced numbers of *Bifidobacterium* spp. were found in infants with an increased risk of developing CD in the whole population and in breast- and formula-fed infants, although breastfeeding favored gut colonization by species of this genus. The findings, therefore, suggest that *Bifidobacterium* spp. and *B. longum* numbers can be influenced by both HLA-DQ genotype and milk-feeding type. Increased numbers of *Staphylococcus* spp. were associated with increased genetic risk of developing CD in the whole population and in both subgroups of breast- and formula-fed infant, and the colonization of this bacterial group was not significantly favored by a specific type of milk-feeding, suggesting that the HLA-DQ genotype plays a more prominent role in the colonization of this bacterial group. In contrast, increased numbers of the *B. fragilis* group were found in infants with high genetic CD risk; however, this association was only confirmed in the subgroup of formula-fed babies but not in those breast-fed, indicating that the type of milk feeding is the main factor influencing the colonization of this bacterial group [55]. A study into the prevalence of *Bacteroides* spp. in a subgroup of this cohort of infants also showed that *B. vulgatus* prevalence was higher in infants at high genetic risk of developing CD, while *B. uniformis* prevalence was increased in infants at low genetic CD risk and in breast-fed infants [54]. Overall, breastfeeding seems to reduce the microbial differences related to the HLA-DQ genotype, which could partly explain the protective effect of human milk reported in some previous observational studies [36, 37]. The followup of this cohort of infants is underway to reveal additional evidence of the role played by early intestinal microbial colonization of the infant's gut in CD development.

Breast milk contains human oligosaccharides (HMOs), which seem to contribute to shaping the composition of the microbiota and selectively stimulate the growth of specific bacterial groups, such as bifidobacteria, in the infant's gut [56]. In breast-fed infants *Bifidobacterium* spp. predominate, representing up to 90% of the total fecal microbiota, whereas in formula-fed infants the microbiota is more heterogeneous and integrated by members of more diverse genera (*Streptococcus*, *Bacteroides*, *Clostridium*, *Bifidobacterium*, etc.) [57, 58]. In recent years, the presence of commensal bacteria in breast-milk of healthy women and their possible transmission to the newborn has also been proposed [59, 60]. The main bacterial genera detected in breast-milk are *Streptococcus* and *Staphylococcus*, representing 36–65% and 29–50% of the total bacteria, respectively, while the genus *Bifidobacterium* only represents 3-4% [61]. However, *Bifidobacterium* spp. becomes predominant in the gut of breast-fed infants, probably due to their better metabolic adaptation to utilizing HMOs as revealed by genome sequencing [62]. The transfer of bifidobacteria from the mother's milk to the infant has been demonstrated by comparing bacterial strains present in mother-infant pair samples [63]. However, the mechanism involved in the colonization on human milk remains unclear. Martín et al. [64] observed different lactic acid bacteria in breast skin and milk, suggesting that these bacteria had a different origin and proposing an endogenous route for breast milk colonization. Meanwhile, *in vitro* studies reported that dendritic cells (DCs) of the lamina propria could penetrate the intestinal epithelium and capture viable bacteria from the lumen, via the opening of the tight junctions between epithelial cells [65]. Commensal bacteria from the mother's gut could use this mechanism to migrate from the intestinal mucosa and colonize the mammary gland. Nevertheless, some animal experiments questioned the complete molecular mechanism involved in this colonization, showing that commensal bacteria in DCs were efficiently killed by macrophages and only pathogens were able to survive in high numbers and during prolonged periods of time [66].

The early colonization of the intestine by the microbiota seems to exert a strong influence on the development of mucosal and systemic immunity, as evidenced by studies conducted with germ-free and colonized mice. In germ-free mice, the serum immunoglobulin levels were reduced and spleens were smaller and the gut-associated lymphoid tissue was immature, with a low content of lamina propria T cells, immunoglobulin (Ig) A producing B cells and intraepithelial T cells; however, these deficiencies were restored after colonization with commensal bacteria [67]. Human studies have also reported that the bacteria colonizing the newborn intestine modifies production of salivary secretory IgA, numbers of circulating IgA and IgM antibody-producing cells as well as the expression of innate immune receptors (e.g., Toll-like receptor) in peripheral blood monocytes [68, 69]. A previous prospective observational study also reports that changes in microbiota composition, characterized by a reduced ratio of *Bifidobacterium* to *Clostridium* counts, early in life precede the development of atopic dermatitis [70], suggesting that the microbiota plays a role in the risk of developing immune-mediated diseases. In this context, other authors [59] also described differences in breast-milk microbiota, characterized by decreased concentrations of *Bifidobacterium* spp. in the milk of allergic mothers compared to healthy mothers, as well as the impact of these differences on the infant's fecal levels of *Bifidobacterium* spp.

## 4. Infections and CD

Infectious agents are considered as possible environmental factors triggering autoimmune diseases. In CD, exposure to infectious agents (viral or bacterial) has also been suggested as a factor causing tissue damage and inflammation, which could eventually contribute to reduced gluten tolerance [71].

Infections can influence the host's immune tolerance by different mechanisms. These include polyclonal lymphocyte activation, increased immunogenicity of organ autoantigens secondary to infection-mediated inflammation, or antigen mimicry molecular mechanisms [72]. In the last case, the autoimmune response is caused by cross-reactivity between host (human) epitopes and immunologically similar epitopes in the infectious organism. The list of such homologies in the bioinformatics-based search is long and can be relevant in some cases. Nonetheless, such homologies do not demonstrate definite evidence for a possible role of the shared antigenic determinant between the infectious agent and the autoantigen [72].

Several studies have described a possible link between CD onset in susceptible patients with diverse infectious agents, and these associations are even traced back to the perinatal period. Several hypotheses about the possible pathogenic mechanisms behind these associations have been discussed in recent years, including antigen mimicry and increased immune activation secondary to infection-mediated inflammation, for example by induction of TNF (tumor necrosis factor)-*α*, IFN-*γ* and IL-15 [73].

In Sweden, a study of 3,392 infants born between 1987–1997, who ultimately developed CD, compared various perinatal data to those of healthy infants and reported that the main risk factor for developing CD was exposure to neonatal infections (OR 1.52; CI 1.19–1.95) [74].

Another study has attempted to establish an association between CD and infections by assessing the serum pathogen-specific antibodies in CD patients compared to the general population [75]. This study examined the association of CD with five major infectious agents, including *Toxoplasma gondii*, rubella virus, cytomegalovirus (CMV), *Treponema pallidum* and Epstein-Barr virus (EBV). The serological results demonstrated a lower prevalence of IgG antibodies in CD patients compared to healthy subjects, suggesting a possible protective role of infections (clinical or subclinical) by EBV, CMV, and rubella on CD development.

One of the first infectious agents associated with CD was the adenovirus type 12 (Ad12). It was proposed that CD could be triggered by the Ad12 infection through mimicry of a peptide within the alpha-gliadin by the Elb protein of this adenovirus 12 [76], but further studies did not support this hypothesis. In one of them, five murine monoclonal antibodies against a synthetic dodecapeptide of *α*-gliadin were tested against whole-wheat gliadin and its subfractions and the prolamins of rye, barley, oats, maize, millet, rice, and sorghum. Four of the antibodies cross reacted with one or more of the celiac nontoxic cereals, and the other did not recognize Frazer's fraction III, a peptic-tryptic digest of wheat-gluten which is known to be toxic [77]. Other studies based on the detection of DNA coding for the E1B-58 kDa protein of this adenovirus on intestinal mucosal biopsies refuted the correlation between CD and active Ad12 infection, although remote infection prior to CD onset could not be excluded [78, 79]. Studies based on detection of antibodies against the virus in serum of CD patients in comparison with controls are also contradictory regarding the association of infection with CD. In a study in favor of this hypothesis, involving 44 children with CD and 60 matched-controls, CD children had significantly higher IgG antibody levels against the Ad12 E1b peptide than the controls [80]. In contrast, another study, including 23 CD patients and 10 control subjects, did not report increased presence of specific antibodies against the E1b-58-kDa protein in serum of CD patients [81].

Another infectious agent epidemiologically associated with CD onset is the hepatitis C virus (HCV). The prevalence of CD in patients with chronic liver disease is 15 times higher than that in the general population [82], and CD is also present in about 5% of the patients with autoimmune hepatic disease [83]. Although HCV has been identified to cause secondary autoimmune processes in other parts of the body, several studies have failed to find increased prevalence of CD in patients with HCV infection [84] and concluded that the association between HCV and CD can be causal or a coincidental finding [85].

Gastrointestinal pathogens like rotavirus have also been linked to CD [86]. The association is based on a prospective study of a cohort of 1,931 children carrying CD HLA risk alleles. This study reported that high frequency of rotavirus infections, estimated by measuring the increase in rotavirus antibodies, may raise the risk of CD autoimmunity, defined as positivity at two or more clinic visits for tissue transglutaminase (tTG) autoantibodies. In addition, there are also studies in which the disease has been associated with *Campylobacter jejuni*, [87] and *Giardia lamblia* [88]. Although some of these reports are case studies based on a single patient, they suggest that gastrointestinal infections may trigger or facilitate the onset of clinical CD by increasing intestinal permeability [3] or by amplifying the immune response to gliadin.

## 5. Involvement of Genetic Factors in CD and Autoimmune Diseases Influencing Immune System Development

As we described previously, the HLA-DQ genes contribute to the 40% of the sibling familial risk of CD and non-HLA linked genes are likely to be the stronger determinants of CD susceptibility [89]. Two complementary approaches have been used to target the genes that account for the remaining 60% genetic linkage and association studies [5]. The genomewide association studies (GWAS) identified a large number of genes involved in CD and other autoimmune diseases outside the HLA region. Some of these genes are IL-2 and IL-21 genes in 4q27 region [3]. In addition, six previously unknown risk regions harboring genes controlling immune responses were identified (CCR3, IL12A, IL18RAP, RGS1, SH2B3, and TAGAP) [90]. Dubois et al. [91] found 13 new genetic risk regions. They described four specific immunological pathways that are relevant to CD pathogenesis, in which genes included in the 13 new risk regions participate. These pathways are involved in (1) T-cell development in thymus, comprising THEMIS, RUNX3, and TNFRSF14 genes; (2) innate immune detection of viral RNA, including TLR8 gene; (3) T- and B-cell costimulation, including CTLA-4, ICOS-CD28, TNFRSF14, CD80, ICOSLG, TNFRSF9, TNFSF4 genes; and (4) production of cytokines, chemokines and their receptors, including TNFSF18 and CCR4 genes.

CD has been associated with autoimmune diseases, particularly with type I diabetes, rheumatoid arthritis, and autoimmune thyroiditis [4, 5]. CD, type I diabetes (T1D), and rheumatoid arthritis (RA) share the HLA region, and other non-HLA regions, including IL2-IL21, SH2B3, and TAGAP regions [92, 93]. CD and T1D have both shown rising incidence in recent years [92], and it has been suggested that gluten consumption, and gut permeability and inflammation are also factors involved in the development of T1D [94]. T-cell infiltration in the target organs, the development of disease-specific autoantibodies, and the role of enzymes involved in posttranslational modifications in the pathogenesis of the diseases are common features in CD and RA although they are distinct in their phenotype [93]. Further studies into the interactions between genetic and environmental factors are necessary to establish the biological mechanisms underlying the development of these diseases and for the design of possible immunomodulatory strategies to interfering some of the common pathologic mechanisms for disease prevention.

## 6. Possible Induction of Acquired Gluten Tolerance

Administration of antigens by the oral route induces hyporesponsiveness to subsequent challenge with the antigen given in an immunogenic form, usually by a parenteral route, a phenomenon termed oral tolerance [95]. Oral tolerance, however, usually affects the response of the local immune system in the intestinal mucosa, thus, preventing hypersensitivity reactions to food proteins that could lead to disorders such as CD or food allergies [96]. Similarly, immunological tolerance prevents the aberrant immune responses to commensal bacteria in the gut [95]. However, the acquisition of oral tolerance is a complex process and is far from being fully elucidated.

Because CD activity is strongly correlated to the presence and dosage of gluten, the induction of gluten-specific oral tolerance is an attractive therapeutic approach. In this line, one population study is currently being carried out to seek new strategies for CD prevention during early of life. PREVENT CD is a project funded under the European Union's FP6, studying the possible induction of gluten tolerance in genetically predisposed children. This is a prospective, randomized, blind dietary intervention in young children from high-risk families for CD (http://www.preventceliacdisease.com/). The rationale is that introducing small amounts of gluten and gradually increasing the amount during a certain timeframe in the infant's development, preferably while they are still being breastfed, will induce oral tolerance to gluten. A total of 1000 children with at least a first-degree relative suffering from CD will participate in this intervention study and followed up for 3 years. From the 4th to 6th months of life they will receive a small amount of gluten, which will be gradually increased during months 6 to 9, and followed by free intake thereafter. The objective of this intervention is to decrease the incidence of CD in the group receiving the gluten supplement compared to the group receiving placebo.

Studies published in the 1980–1990s led to the idea that the mechanisms responsible for oral tolerance depend on the feeding pattern, which can induce tolerance by either clonal anergy (or deletion) of specific T cells or by the induction of regulatory T (Treg) cell activity [97]. More recent knowledge has pointed out antigen presenting cells (APCs) as fundamental players directing tolerance or immunity towards specific antigens.

Generation of immunological tolerance has been attempted through ingested or inhaled soluble proteins via the oral and nasal routes [98]. An attempt was made to induce gliadin tolerance in HLA-DQ8 transgenic mice immunized by intra foot pad injections of gliadin in Freund's adjuvant, after soluble gliadin had previously been instilled into their nostrils following a tolerization protocol. A decrease in systemic T cell responses to the recombinant *α*-gliadin was found, as reflected by a lymphocyte proliferation assay. While the immunization protocol induced transcription of both Th1 and Th2 cytokines, the tolerization protocol only significantly down-regulated IFN-*γ* mRNA expression [99]. This finding highlights the potential use of this strategy for the immunomodulation of this disease. However, the presence of gliadin-specific T cell clones in transgenic mice models is not sufficient to develop enteropathy and, therefore, the down-regulation of these clones might not be sufficient alone to control the disease, necessitating the control of other pathogenic pathways involved in CD.

DCs play a central role in intestinal immune responses to dietary proteins and commensal microbiota. Current evidence suggests that tolerance requires migration of DCs that have taken up antigens in the mucosal lamina propria to the mesenteric lymph nodes (MLNs) [100]. On the other hand, the production of TGF-*β* and retinoic acid (RA) by DCs drives the development of Treg cells. IL-15 reduces the ability of DCs to induce the generation of Treg cells and, unexpectedly, RA synergizes with IL-15 in reducing the number of Treg cells [101]. A double transgenic murine model partially reproducing human CD was used to demonstrate that RA synergizes with IL-15 in promoting gluten tolerance breakdown by inducing DC-mediated production of proinflammatory cytokines (IL-12 and IL-23) and a Th1 response to gluten [101]. Therefore, RA is not only a mediator of tolerance (as previously thought), but in the presence of other cytokines it may activate DCs towards a proinflammatory phenotype and induce specific proinflammatory Th1 responses. Therefore, future immune therapies based on RA, aiming to restore oral tolerance, should be employed with caution because the presence of IL-15 (and/or other proinflammatory cytokines) may also have undesirable effects.

 With respect to restoring immune tolerance, it is worth mentioning the development of a peptide-based therapeutic vaccine, which has induced immune tolerance in rodent models of HLA-DQ2-restricted T-cell immunity to gluten [102]. The safety of this preparation (a combination of toxic peptides termed Nexvax2*; *Nexpep, Ivanhoe, Vic, Australia), aimed at inducing desensitization as traditional vaccines do, is currently under investigation in patients with treated CD.

Active delivery of recombinant autoantigens or allergens at the intestinal mucosa by genetically modified *Lactococcus lactis* has also been proposed as a novel therapeutic approach for tolerance induction. *L. lactis* was genetically engineered to secrete a deamidated DQ8-specific gliadin epitope (DQ8d), which is immunodominant for DQ8-mediated T-cell responses (LL-eDQ8d). Its oral supplementation to NOD AB° DQ8 transgenic mice (using the NOD background, which contributes to autoimmunity and pathogenesis, in combination with expression of a human DQ8 MHC class II transgene, which contributes to sensitivity to gliadin) was studied [103]. This treatment resulted in an Ag-specific decrease of the proliferative capacity of inguinal lymph node cells and lamina propria cells. Production of IL-10 and TGF-*β* and a significant induction of Foxp3^+^ regulatory T cells were associated with the eDQ8d-specific suppression induced by LL-eDQ8d [103]. These data provide support for the development of effective therapeutic approaches for gluten-sensitive disorders using orally administered Ag-secreting *L. lactis*.

The studies showing that intestinal dysbiosis is associated with CD and that there are differences in the intestinal colonization pattern of infants with different genetic risk of developing CD and under different types of milk-feeding, would suggest the potential use of interventions with probiotics in the gut ecosystem to promoting the development or increasing gluten tolerance. So far, *B. longum* CECT 7347 (IATA-ES1) and *B. bifidum* CECT 7365 (IATA-ES2) have been shown to reduce inflammatory cytokine production (IFN-*γ* and TNF-*α*) induced by the altered microbiota of CD patients, and to increase IL-10 production by peripheral blood mononuclear cells *in vitro* [104]. These bifidobacterial strains also provide protection against inflammation and mucosal damage triggered by gliadin peptides, combined or not with IFN-*γ*, in Caco-2 cultures [105] and in intestinal loops of germ-free rats [105, 106]. In intestinal loops, *B. bifidum* CECT 7365 increased the number of goblet cells in the small intestine, which were reduced by the presence of gliadin, IFN-*γ* and enterobacteria, and upregulated ZO-1 expression, which was downregulated by CD triggers (gliadin peptides and IFN-*γ*) [106]. *B. bifidum* CECT 7365 also increased the production of inhibitors of metalloproteinases (TIMP-1), which could contribute to gut mucosal protection [106]. In an animal model of this enteropathy, induced by oral administration of gliadin to neonatal rats sensitized with IFN-*γ*, administration of* B. longum* CECT 7347 attenuated inflammatory cytokine (TNF-*α*) production and induced IL-10 production in the small intestine, and also attenuated the CD4+ T-cell mediated immune response in peripheral blood. In contrast, *Bifidobacterium lactis* NCCC2818 and several strains of *Lactobacillus* spp. (*L. plantarum* ITM21B, *L. paracasei* IMPC2.1,* L. fermentum* DRL38, and *L. casei* ATCC 9595) exerted inductive rather than suppressive effects on both the innate and adaptive immunity in cell cultures *in vitro* and *in vivo* in DQ8 transgenic mice administered gliadin intragastrically and cholera toxin as adjuvant [107]. The effects of the strains on immature bone marrow-derived DCs, extracted from these transgenic mice, were analyzed *in vitro*. Results showed that all strains increased CD86 expression, indicative of DC maturation, none of the strains induced IL-10 or IL-12 production, while *L. paracasei* IMPC2.1 and *L. fermentum* DRL38 increased TNF-*α* expression. Moreover, *in vivo* administration of these strains to the same DQ8 transgenic mouse model enhanced the antigen-specific TNF-*α* secretion. In the same DQ8 mouse model, *in vivo* administration of *L. casei* ATCC 9595 increased the gliadin-specific response mediated by CD4(+) T cells, increasing the gliadin-specific IFN-*γ* expression and proinflammatory polarization of the cytokine milieu in the small intestinal mucosa. In the light of these results, it was suggested that *L. casei* ATCC 9595 could be used as vaccine adjuvant to enhance both mucosal and systemic T cell-mediated responses to gluten [108]. Subsequent studies were conducted in the same DQ8 transgenic mice, which were intragastrically administered gliadin and cholera toxin as an adjuvant and were also given indomethacin orally to induce the tissue lesions. Results showed that *L. casei* ATCC 9595 restored intestinal damage and TNF-*α* production, but positive effects on gliadin-specific production of IFN-*γ*, IL-10, and IL-4 were not detected [109]. However, the animal models and experimental conditions used were not comparable, thus definitive conclusions cannot be drawn about the differential effects of different bacterial strains. Moreover, further animal studies focusing on CD prevention would be required to sustain the notion that specific components of the gut microbiota can contribute to the development of gluten tolerance in early life.

In addition, human studies would be essential to definitively conclude that specific bacteria play a protective role in CD patients, or susceptible individuals, by modulating immune responses and inducing or increasing tolerance to gluten.
